# The effects of *Euphorbia hirta* on the ultrastructure of the murine liver, kidney and aorta

**DOI:** 10.3892/etm.2013.1295

**Published:** 2013-09-13

**Authors:** J.Y.R. WONG, Y.S. CHEN, S. CHAKRAVARTHI, J.P. JUDSON, SANTHANA RAJ L., H.M. ER

**Affiliations:** 1 Division of Human Biology, International Medical University, Kuala Lumpur 57000;; 2 Division of Pathology, International Medical University, Kuala Lumpur 57000;; 3 Electron Microscopy Unit, Institute for Medical Research, Kuala Lumpur 50588;; 4 Department of Pharmaceutical Chemistry, International Medical University, Kuala Lumpur 57000, Malaysia

**Keywords:** *Euphorbia hirta*, ultrastructure, aorta, kidneys, liver

## Abstract

*Euphorbia hirta* is widely used in traditional remedies and has been used cross-culturally for generations against maladies such as asthma, skin ailments and hypertension. Previous studies have demonstrated that *Euphorbia hirta* has antibacterial activity, and have also indicated certain antimolluscidal, antimalarial and anti-inflammatory properties, the latter of which have been suggested to be more pronounced than those of the rheumatological drug, etanercept. To date, no studies have identified the anatomical effects of this herb on the organs of test animals. This study aimed to identify the effects of *Euphorbia hirta* on the ultrastructure of the murine liver, kidney and aorta. A total of 32 adult male Sprague-Dawley rats were divided into four groups; three groups were fed with aqueous extracts of *Euphorbia hirta* at doses of 1, 10 and 50 mg/kg, respectively, every alternate day for 50 days, while one group served as a control. The animals were later sacrificed and the liver, kidney and aorta harvested for examination by electron microscopy. The aorta showed no ultrastructural changes across the groups. Renal and hepatic tissue from the treated groups demonstrated dose-dependent injuries, which showed architectural damage beginning in the nuclei and spreading outwards. Taking into consideration the properties of *Euphorbia hirta* that have been described in previous studies, in addition to the results from the present study, it appears that the herb may exhibit similar effects to those of the quinolone group of antibiotics. Further in-depth investigations are required into the potential effects of *Euphorbia hirta*, deleterious and otherwise.

## Introduction

*Euphorbia hirta* is otherwise known as the Australian asthma herb, *fei yang tsao* in China, *dudhi* in India, *gelang susu* in Malaysia, *patikan kerbau* in Indonesia and *tawa-tawa* or *gatas-gatas* in the Philippines, and is included in traditional remedies in numerous cultures (1–3). *Euphorbia hirta* belongs to the *Euphorbiaceae* family. Visually, the plant displays purple flowers and produces white, creamy latex when its stem is split (4,5). Its leaves grow in opposition to each other and are short and oblong shaped. The edges of the leaves are serrated and they exhibit a purple splotch down the middle (2). However, regional differences in the appearance of the plant have been noted.

Analyses of *Euphorbia hirta* extracts have revealed the presence of chemicals, including tannins, phenols, flavanoids, butanol and alkaloids (2,6–8). These components have been hypothesised to be responsible for the properties shown by the plant.

Traditionally the plant has been applied as a poultice to wounds and cuts (5,9), while skin ailments, such as warts, boils and abscesses, have also been treated using *Euphorbia hirta* (5,9). Furthermore, *Euphorbia hirta* has been used to treat dysentery, gonorrhoea and conjunctivitis (10) and more severe illnesses, such as malaria, have apparently shown improvement with consumption of the herb (11). In addition, male sexual dysfunction is remedied by Nigerian traditional healers using *Euphorbia hirta* (12), while Swahili and Sukuma individuals treat hypertension and oedema using this herb (13).

*Euphorbia hirta* has been shown to possess potent antimicrobial, anti-inflammatory and antihelminthic activities. Its anti-inflammatory properties have demonstrated efficacy against rheumatoid arthritis in Ncf1^DA^ arthritis-prone rats, superseding that of etanercept, which is a tumour necrosis factor α (TNF-α) inhibitor currently used as a frontline disease-modifying anti-rheumatic drug (14). Ethanolic and aqueous leaf extracts of *Euphorbia hirta* have shown diuretic effects similar to those of acetazolamide when tested on rats (13). Furthermore, the blood glucose levels of albino mice with streptozocin-induced diabetes have also demonstrated improvements following treatment using aqueous extracts of the *Euphorbia hirta* (15) and the herb has been shown to reduce the gastrointestinal motility of rats with castor oil-induced diarrhoea (16).

There has been comparatively little investigation into the potential side-effects of *Euphorbia hirta* consumption. Adedapo *et al* (17,18) described an increase in serum biomarkers for liver [aspartate aminotransferase (AST) and alanine aminotransferase (ALT)] and renal (creatinine and urea) function, as well as leucocytosis and uraemia in rats that were force-fed with *Euphorbia hirta* extracts. Furthermore, Chee (19) demonstrated an increase in the contractility of isolated thoracic rat aortae *in vitro* with respect to phenylephrine.

The aim of this study was to examine the effects of aqueous extracts of *Euphorbia hirta* on the ultrastructure of the murine liver, kidney and aorta.

## Material and methods

### Materials

#### Plant material

Raw *Euphorbia hirta* was procured from Taiwan and authenticated by Ron Vickery, Department of Botany, Natural History Museum of London (UK).

#### Animals

Thirty-two adult male Sprague-Dawley rats, weighing 300–500 g, were used in this study. All rats were purchased from Universiti Putra Malaysia (UPM; Selangor, Malaysia). The rats were housed in the Animal Holding Facility of the International Medical University (IMU; Kuala Lumpur, Malaysia) which was maintained at a constant room temperature of 24°C with a 12-h dark/light cycle. Reverse osmosis water and standard rat feed (Perniagaan Usaha Cahaya, Serdang, Malaysia) were provided *ad libitum*. Prior to commencing the feeding of the rats with *Euphorbia hirta* extracts every alternate day for 50 days, a one-week adjustment period was allotted. Experimental procedures were reviewed, monitored and approved by the Research and Ethics Committee of the IMU.

### Methods

#### Preparation of extract

Powdered *Euphorbia hirta* (30 g) was sequentially extracted with 300 ml of each n-hexane, chloroform, methanol and water. A total of 90 g powdered *Euphorbia hirta* was used in the preparation of the extract.

The solvents in the extracts were removed via rotary evaporation under reduced pressure. The aqueous extract was further freeze dried and stored at 4°C. The yield of the aqueous extract was 9%, based on the dry weight of the plant.

#### Animal feeding

The male Sprague-Dawley rats were divided into four groups, with eight rats assigned to each group. Every cage housed four rats, which were tagged on their tails. Group 1 was fed phosphate-buffered saline (PBS) as a control, while Groups 2-4 were fed with increasing concentrations of aqueous extracts of *Euphorbia hirta*, at 1, 10 and 50 mg/kg, respectively.

All animals were weighed daily. The rats were fed the extracts using oral gavage and the standard volume of feed was 1 ml. The rats received the extracts or PBS every alternate day for a period of 50 days prior to the animals being sacrificed via cardiac puncture.

#### Organ harvesting and processing for storage

The thoracic aorta, defined as the section of the aorta stretching from the end of the arch of the aorta to the diaphragm, was used in this study. The perivascular adipose tissue (PVAT) was completely stripped off. Similarly, the livers and kidneys were harvested and the surrounding fatty tissue was removed.

A single aortic ring was apportioned out and sizable portions of the liver and kidneys were removed; all were placed in 2.5% glutaraldehyde in 0.1 M sodium cacodylate buffer. The aortic ring was then sectioned into smaller rings, with a maximum width of 1 mm^3^, and the liver and kidney were manually sectioned into cubes, also measuring ≤1 mm^3^. All sectioning was performed whilst the organs were soaked in 2.5% glutaraldehyde in 0.1 M sodium cacodylate buffer.

Saline (9 g NaCl per 1 litre H2O) was used to rinse the sectioned tissues, prior to the tissues being placed in 2.5% glutaraldehyde in 0.1 M sodium cacodylate buffer and stored at 4°C. A minimum ratio of 1:17 (tissue to buffer volume) was maintained to ensure hydration.

#### Tissue processing for electron microscopy

Sample processing was conducted in the Electron Microscopy Unit of the Institute of Medical Research (Kuala Lumpur, Malaysia). Tissue samples were embedded in epoxy resin using a set protocol from the University of Bristol (20). The embedded samples were then sectioned into ultrathin slices using glass knives at a thickness of 90 nm.

## Results

Electron microscopy was performed to view the changes that may otherwise not have been visible with light microscopy or the naked eye. Minute changes conferred reasonable confidence into the pathological processes that may have taken place.

### 

#### Aorta

**Figure 1.**
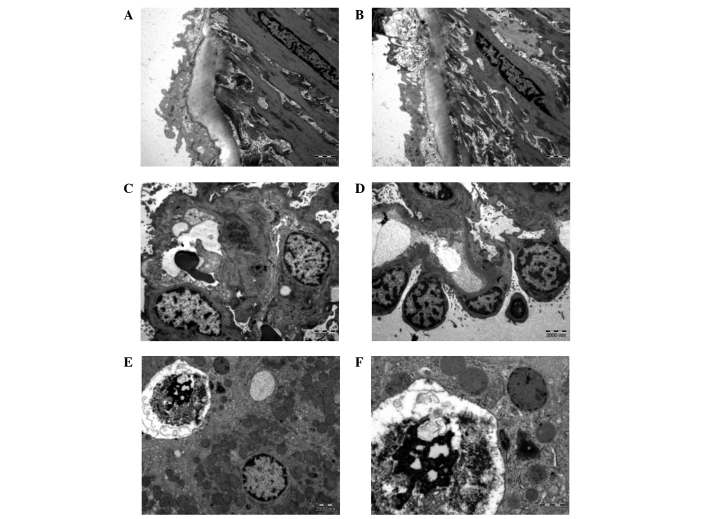
Tunica intima of an aorta from (A) the control group (magnification, ×13,500) and (B) group 2 (magnification, ×13,500). (C) Glomerulus with thickened basement membrane and near-normal podocytic nuclei, which are irregular with appreciable euchromatin (group 2; magnification, ×4,400). (D) The few podocytes here show much euchromatin clumping together, probably demonstrating early stage pyknosis. The basement membrane is also thickened (group 2; magnification, ×4,400). (E) The top left hand corner shows a degenerated hepatocyte from group 4. The nuclear material has completely condensed. A vacuole may be observed next to the degenerated hepatocyte, as well as a healthy hepatocyte at the bottom right (magnification, ×4,400). (F) Close up view of a degenerated nucleus from group 4 (magnification, ×11,000). Group 1, control group, fed with phosphate-buffered saline; groups 2 and 4, treatment groups, fed with 1 and 50 mg/kg *Euphorbia hirta*, respectively.

The ultrastructure of the aorta showed no change in the endothelium, smooth muscle and elastin of the treated groups when compared with those in the control group. Photomicrographs of the aorta are shown in Fig. 1A and B.

#### Kidneys

Glomeruli of the control group displayed features typical to those of a normal glomerulus. The basement membranes were intact without any signs of damage, while the podocytes and mesangial cells were observed to be healthy. All treated groups displayed signs of damage to the basement membrane. Podocytes and mesangial cells showed changes to their nuclei, such as pyknosis and karyolysis, with the damage appearing to be dose-dependent. These photomicrographs are shown in Fig. 1C and D.

#### Liver

All liver samples viewed under the electron microscope showed the clear presence of hepatocytes. The control group hepatocytes possessed the typical features of a normal hepatocyte. By contrast, the hepatocytes in the treated groups were in various stages of degeneration. These included a marked reduction in the size of the nucleus, chromatin condensation (pyknosis) and nuclear fragmentation (karyorrhexis). The changes appeared to be dose-dependent, consistent with the changes in the kidney. Photomicrographs of the liver are shown in Fig. 1E and F.

## Discussion

To the best of the authors’ knowledge, there have not been any studies, to date, in which similar methods have been used to identify the side-effects of *Euphorbia hirta*. Therefore, further studies concerning this issue are warranted.

The results obtained from the present study demonstrated a dose-dependent effect of *Euphorbia hirta* on the murine liver and kidneys. Consolidation of these data and the various other properties of the herb described in previous studies appear to suggest that *Euphorbia hirta* exhibits pharmacological activities similar to those of quinolones, a group of broad-spectrum antibiotics. A number of examples are described below.

Oxolinic acid, a first generation quinolone, has been shown to exert stimulant effects on mice, due to the properties that classify it as a dopamine reuptake inhibitor (21). This stimulant effect is consistent with the findings of induced stress reduction studies using *Euphorbia hirta* (22–24). A common interaction site, the γ-aminobutyric acid A (GABA_A_) receptor Cl^−^ complex, has been identified (25,26). Moxifloxacin, a fourth generation synthetic fluoroquinolone, has been contra-indicated in patients suffering from myasthenia gravis, which is in accordance with the apparent ability of *Euphorbia hirta* to restore acetylcholinesterase activity (23,27). Interestingly, the quinolones are associated with the quinoline family, as are mefloquine and lariam, a synthetic analogue of quinine (11,28). This correlates with the antimalarial activity of *Euphorbia hirta*. Tosufloxacin, a fluoroquinolone, has been indicated to cause severe nephritis (29), while there have been numerous case reports revealing renal toxicity issues associated with norfloxacin, such as nephritic syndrome, renal failure and acute interstitial nephritis (30–33). Furthermore, there were sufficient postmarketing complaints concerning renal dysfunction and hepatotoxicity following the use of temafloxacin for it to be withdrawn from the market (29,34).

Electron microscopy isolates minute portions of sample organs, which may not be reflective of the global condition of the organ. Whilst in the current study, isolated focal areas of damage were isolated, this does not imply that organ integrity and function were compromised. This study is not able to confirm or deny the presence of compromised organ function. It is merely an indication of the consequence of regular *Euphorbia hirta* consumption.

Current medical interpretation techniques focus on histopathology and biochemistry, as well as the correlation with clinical signs and symptoms. While our results demonstrated damage to the murine organs with *Euphorbia hirta* use, the use of enzyme biomarkers for the respective organs, i.e., AST and ALT for the liver and urea and creatinine for the kidneys, is likely to validate our results further.

There are relatively few articles that discuss the negative effects of any herbal remedy while at the same time utilising modern pathological techniques. This is probably not due to disinterest, rather to the immense difficulty of obtaining verified data on the usage and consumption of herbal remedies across communities. To complicate matters, the majority of remedies are passed on by word of mouth. As a result, there are not many studies with which to compare our results.

Despite the limitations faced, our results indicate that *Euphorbia hirta* has a certain ill effects on the end consumer. The proposed association between quinolones and *Euphorbia hirta* is premature and may prove to be incidental. In conclusion, this study is indicative of the effects of *Euphorbia hirta*; however, it should not be used to predict the effects of the herb. To date, the authors have not uncovered any study using similar methods to identify the side-effects of *Euphorbia hirta*. Therefore, further studies are required.

The results revealed in the present study, which indicate that the consumption of *Euphorbia hirta* may be associated with side-effects, warrant further investigations. Moreover, a further detailed analysis of the herb’s biochemistry is required.
